# Trend of determinants of birth interval dynamics in Bangladesh

**DOI:** 10.1186/s12889-016-3577-9

**Published:** 2016-09-05

**Authors:** Jahidur Rahman Khan, Wasimul Bari, A. H. M. Mahbub Latif

**Affiliations:** 1 Institute of Statistical Research and Training (ISRT), University of Dhaka, Dhaka, 1000 Bangladesh; 2 Department of Statistics, Biostatistics & Informatics, University of Dhaka, Dhaka, 1000 Bangladesh; 3 Center for Clinical Epidemiology, St. Luke’s International University, 3-6-2 Tsukiji, Chuo-ku, Tokyo, 104-0045 Japan

**Keywords:** Fertility, Survival model, Correlated time-to-event data, Demographic health survey

## Abstract

**Background:**

The distribution of birth intervals can be used to draw attention to important characteristics of dynamics of fertility process. The main objective of this paper is to examine the effects of socioeconomic, demographic and proximate determinants on the length of birth intervals of women of Bangladesh and also to see whether the effects are changed over the years.

**Methods:**

Birth intervals can be considered as correlated time-to-event data because two or more birth intervals could correspond to a single mother. Moreover, women from the same neighborhood usually share certain unobserved characteristics, which may also lead to correlated time-to-event data (birth interval). A parametric random effect (frailty) model is used to analyze correlated birth interval data obtained from three Bangladesh Demographic and Health Surveys (BDHS 2004, 2007, and 2011).

**Results:**

The results show that alongside different socioeconomic, demographic determinants, unobserved community and mother effects have considerable impact on birth interval in Bangladesh. However, the effects of different factors on birth interval changes in a small scale over the duration of 2004–2011.

**Conclusions:**

Efficient policy is a priority for promoting longer birth spacing and achieving a decline in fertility.

## Background

Fertility is a principal component of population dynamics that determines the structure of the population of a country. Analysis of birth interval (time between two successive live births) is more susceptible method for measuring fertility compared to the other methods [1, 2] and it can provide further insight into the mechanisms of underlying changes in the fertility. In developing countries, birth interval analysis is preferred over analyzing the number of children ever born to women (completed parity), especially if urgent results are required for fertility consideration. As a developing country of South-Asia, Bangladesh faces a number of major challenges, e.g. excessive population, which is about 160 million. Over the study period (i.e. from the year 2004 to 2011), Bangladesh is performing well in controlling the population growth, for instance, total fertility rate (TFR) reduced from 3.0 to 2.3 and median birth interval increased from 39 to 47 months [3]. Bangladesh needs more effort on controlling population size because the recent improvement has not reach to a reasonable level yet compared to the other developing countries in the world.

Birth intervals can be considered as correlated time-to-event data because a mother could experience repeated occurrence of the same event (childbirth). Duration between two successive live births, and that is between the last live birth and the interview date are considered as closed (event time) and open (censoring time) birth intervals, respectively.

Among the very few published papers on birth interval dynamics in Bangladesh, Zenger [4] studied siblings neonatal mortality risks and birth spacing in Bangladesh, and Chakraborty et al. [5] studied the differential patterns of birth interval in Bangladesh by identifying the important factors contributing to the length of birth intervals. However, birth intervals obtained from the same mother were considered as independent in most of the studies [6, 7]. Majority of demographic and health surveys of the developing world use multi-stage cluster sampling design, where clusters often consist of 100–300 neighboring households. Birth intervals within a cluster can be assumed to be correlated because mothers from the same geographical cluster could share certain type of unobserved environmental factors. Modeling birth intervals without considering within mother and/or within cluster correlation may lead to incorrect standard errors of the estimators of model parameters [8]. Recently, Mahmood et al. [9] analyzed birth intervals of Bangladeshi women after simultaneously adjusting the heterogeneity due to cluster and mother. Among the papers published in demographic literature on the analysis of correlated time-to-event data, Vaupel et al. [10] used random effect or frailty in the context of mortality studies, and Guo and Rodriquez [11] presented a multivariate proportional hazards model with the single frailty term. A detail discussion on extending the univariate survival models for modeling dependence in multivariate or correlated time-to-event data can be found in [12, 13].

Different demographic and socioeconomic characteristics are found to be associated with the distribution of the length of birth interval. For example, mother’s age at the birth and the birth order (parity) of the index child have potentially different effects on birth intervals [9]. Survival status of the index child and maternal education have influence on the birth interval [9, 14, 15]. In developing countries, preference for a balanced family (at least one child of each sex) is more prevalent, where son preference is most comprehensive in South-East Asia [16–18]. So, family composition of children is a potential factor for determining the birth spacing [9, 14]. According to Nath et al. [2], wealth index of family has an effect on the birth interval. Peoples’ culture, lifestyle and socioeconomic conditions may differ according to place of residence (rural and urban) and administrative divisions, which may play vital role on the distribution of birth interval [19, 20].

The main objective of this paper is to identify important factors for the distribution of the length of time intervals and also to examine whether these effects are changed over time. This will help understanding the birth interval dynamics as well. Two different parametric frailty models have been considered for modeling the birth interval: one models cluster effects in mother and another in community level to explore the unobserved heterogeneity. The paper is organized as follows: Section “Methods” describes the methodology of parametric frailty model, Section “Results” contains the description of data and results of the fitted model, Section “Discussion” contains the detail discussion of results, and Section “Conclusions” contains a brief conclusion.

## Methods

### Parametric frailty models

Let *t*_*ij*_ be the birth interval corresponding to the *j*^*th*^ child in the *i*^*th*^ cluster and *δ*_*ij*_ is the corresponding censoring indicator (*i*=1,…,*K*; *j*=1,…,*n*_*i*_). For the *j*^*th*^ subject of the *i*^*th*^ cluster, the hazard function at time *t* is defined according to a proportional hazards model with a random effect (frailty) as

1 $$\begin{array}{*{20}l} h_{ij}(t \,|\, X_{ij},u_{i})=h_{0}(t)u_{i}\exp({\beta}'X_{ij}),  \end{array} $$

where *h*_0_(*t*) is the baseline hazard function, *u*_*i*_ is the unobserved frailty term, and *X*_*ij*_ is the *p*-dimensional vector of covariates and *β* is the corresponding *p*-dimensional vector of regression coefficients.

As in the proportional hazards model, the form of the baseline hazard function can either be unspecified or be modeled using a parametric function. The method of estimating frailty model (1) depends on the form of the baseline hazard function assumed. For unspecified baseline hazard function, the Expectation-Maximization algorithm is used to optimize the corresponding likelihood function with frailties (*u*’s) are considered as missing observations. On the other hand, for some specific choices of parametric model for the baseline hazrd function, the frailty model (1) can be estimated by maximizing the corresponding marginal log-likelihood function. The parametric estimation will be more powerful and simpler if the form of baseline hazard function is known in advance, where as semi-parametric approach offers more flexibility in estimation. Different parametric distributions can be considered for the baseline hazard function. Morris et al. [21] and Pickles et al. [22] used Weibull distribution for baseline hazard function. Weibull model is flexible in describing time-to-event data and its shape parameter allows for different shapes of the hazard function. In this paper, Weibull model is assumed for baseline hazard function, *h*_0_(*t*)=*γt*^*γ*−1^/*α*^*γ*^, *α*>0, *γ*>0. The parametric baseline hazard function not only makes the estimation of model parameters easier, but also describes explicitly the effect of the frailty on hazard ratios over time.

The choice of the frailty distribution is very important and different frailty models have been suggested in the literature [13, 23]. Though there is no hard and fast rule of selecting a frailty distribution, gamma distribution is most commonly used frailty model. This is due to mathematical convenience [23] rather than of scientific motivation, e.g. choosing a gamma distribution for the frailty corresponds to a simpler expression of the likelihood function. In this paper, the following one-parameter gamma distribution is assumed for the frailty

2 $$\begin{array}{*{20}l} g(u;\theta)=\frac{u^{\frac{1}{\theta}-1}\;\exp(-u/\theta)}{\theta^{\frac{1}{\theta}}\;\Gamma{(1/\theta)}},\; \theta>0,  \end{array} $$

with *E*(*U*)=1 and *Var*(*U*)=*θ*. The frailty distribution parameter *θ* provides information on the variability of the population of clusters and a large value of *θ* indicates that there is a higher degree of heterogeneity among the clusters, and a strong association among the observations within a cluster. This association can be quantified by Kendall’s tau, which can be expressed as a function of frailty distribution parameter *θ* as *τ*=*θ*/(*θ*+2).

### Estimation of model parameters

In the parametric setting, estimation of parameters is based on the marginal likelihood in which the frailty terms have been integrated out by averaging the conditional likelihood with respect to the distribution of frailty. Condition on the frailty *u*_*i*_, the likelihood function for the *i*^*th*^ cluster is then given by

3 $$ \begin{aligned} L_{i}({\phi},{\beta} \,|\, u_{i})&=\prod_{j=1}^{n_{i}} h_{ij}(t \,|\, Xij, ui)^{\delta_{ij}}\\ &\quad \times S_{ij}(t \,|\, Xij, ui),  \end{aligned}  $$

where the hazard function *h*_*ij*_(· | ·) is defined in (1), the corresponding survival function is $S_{ij}(t \,|\, Xij, ui)=\exp \left \{-{\int _{0}^{t}} h_{ij}(x \,|\, Xij, ui)dx\right \}=\exp \left (-H_{0}(t)u_{i}{\beta }'Xij, ui\right)$ with $H_{0}(t)={\int _{0}^{t}} h_{0}(x)dx$ is the cumulative baseline hazard function, and the baseline hazard function *h*_0_(*t*) has already been is defined as a function of the vector of parameters *ϕ*=(*α*,*γ*)^′^. It follows that, the marginal likelihood function for the *i*^*th*^ cluster is

4 $$ \begin{aligned} L_{m,i}({\phi},\theta,{\beta})&=\prod_{j=1}^{n_{i}}\int_{0}^{\infty}\left[h_{0}(t_{ij})u_{i}\exp({\beta}'Xij, ui)\right]^{\delta_{ij}}\\ &\quad\times S_{ij}(t_{ij}\,|\, Xij, ui)g(u_{i};\theta)\,du_{i}. \end{aligned}  $$

Taking the logarithm of the expression the *L*_*m*,*i*_ in (4) and summing over the *K* clusters, the marginal log-likelihood function can be obtained [24], which takes the following form

5 $$  \begin{aligned} l_{m}({\phi},\theta,{\beta})&=\sum_{i=1}^{K}\left[\log\Gamma\{(1/\theta)+d_{i}\}+\sum_{j=1}^{n_{i}}\delta_{ij}\right.\\ &\quad\times\left({\beta}'Xij, ui+\log\!\left(\gamma t_{ij}^{\gamma-1}/\alpha^{\gamma}\right)\!\right)\,-\,\log \Gamma(1/\theta)\\ &\quad+d_{i}\log\theta-\{(1/\theta)+d_{i}\}\\&\left.\quad\log\left(1+\theta\sum_{j=1}^{n_{i}}(t_{ij}/\alpha)^{\gamma}\exp({\beta}'Xij, ui)\right)\right], \end{aligned}  $$

where $d_{i}=\sum _{j=1}^{n_{i}} \delta _{ij}$ defines the total number of events observed in the *i*^*th*^ cluster. The maximum likelihood estimators of (*ϕ*,*θ*,*β*) correspond to the maximum of the marginal log-likelihood function *l*_*m*_ is defined in (5). The asymptotic variance-covariance matrix of the maximum likelihood estimators can also be easily derived from the corresponding information matrix.

### Data and variables

Birth interval data obtained from the Bangladesh Demographic and Health Surveys (BDHSs) of the years 2004, 2007 and 2011 have been analyzed in this paper. Demographic and Health Surveys (DHSs) have been conducted in many developing countries of the world for some years now, and different national and sub-national indicators obtained from such surveys have been used by the respective governments for planning short- and long-term policies. These surveys are implemented through a joint efforts of the respective government, Macro International, and USAID, and a multistage stratified cluster sampling was used to select sampling units for each of these surveys. For Bangladesh, each of the administrative divisions is divided into 12 strata (rural-urban by division) and each of the stratum consists of a number of clusters. At the first stage of the sampling, 361 clusters were selected from the available strata and in the second stage, about 30 households were selected from each of the selected clusters using a systematic random sampling method. A total of 10,523, 10,461 and 17,511 households were occupied in the samples from the sampled households of the years 2004, 2007, and 2011, respectively. A total of 11,440, 10,996, and 11,832 ever-married women from the sampled households were interviewed to collect data for the years 2004, 2007, and 2011, respectively. Information on fertility, awareness of and use of family planning methods, nutritional status of women and young children, childhood mortality, maternal and child health, among others were collected from these surveys.

In this study, women who have at least one birth in preceding five years of the respective survey years were selected from the BDHS (2004, 2007, 2011) data and all birth intervals corresponding to the selected women were used in the analysis. As mentioned in the Section 1, birth intervals could be either open-ended (duration between last birth and interview date) or closed (duration between two successive live births). By considering open-ended and closed birth intervals as censored and event time, respectively, birth intervals are considered as time-to-event data in this paper. Among the important covariates for modeling birth intervals that were reported in earlier studies [1, 9], those available in BDHS data were selected for this study. Mother’s age at the first birth of the child, parity, family composition (“girl”, “boy”, “girl, boy”, “girl, girl”, “boy, boy”), survival status of the index child (“death”, “alive”), mother’s education (“no education”, “primary”, “secondary”, “higher”) were considered potentially important factors in analyzing birth spacing and were included in the analysis. Wealth index was calculated using principle component analysis of asset variables, which is categorized as (“poorest”, “poorer”, “middle”, “richer”, “richest”).

## Results

### Analysis of birth interval in Bangladesh

#### Exploratory analysis

Tables 1, 2 and 3 provide descriptive statistics of the covariates selected for modeling birth interval for the years 2004–2011. All the descriptive statistics are reported by different values of parity. The results show that the percentage of mothers with three or more children decreased about 10 % (45 to 35 %) over the years 2004–2011. On the other hand, the percentage of mothers with exactly two children increased about 4 % (26 to 30 %) over the years 2004–2011. For mothers, the average age at the first birth increased from 17.5 years to 18.1 years over the survey years. The results show that the proportion of illiterate mothers decreased from 34 to 18 % and that of with secondary or higher education increased from 35 to 52 % over the years 2004–2011, and more than half of the higher educated mothers had one child. Moreover, the proportion of deaths of the index child during infancy decreased gradually from 9 to 6 % over the years 2004 to 2011.

**Table 1:** Descriptive statistics of selected covariates by parity for BDHS, 2004

	1	2	3	4	>4	Overall
Family composition						
(Boy)	.515(721)					.153(721)
(Girl)	.485(678)					.144(678)
(Boy, Girl)		.509(609)				.130(609)
(Boy, Boy)		.252(302)				.064(302)
(Girl, Girl)		.239(286)				.061(286)
(>2 children)						.448(2105)
Survival status						
Died						.088(1145)
Mother’s age at first birth						
Mean ± SD						17.5 ±3.2
Median						17.0
Mother’s Education						
No education	.123(198)	.190(305)	.221(355)	.170(273)	.296(475)	.342(1606)
Primary	.262(378)	.274(396)	.197(284)	.123(177)	.145(209)	.307(1444)
Secondary	.481(646)	.296(397)	.132(177)	.050(67)	.041(55)	.285(1342)
Higher	.573(177)	.320(99)	.087(27)	.019(6)	.000(0)	.066(309)
Wealth quintile						
Poorest	.198(206)	.210(219)	.189(197)	.155(161)	.249(259)	.222(1042)
Poorer	.273(239)	.235(206)	.177(155)	.115(101)	.199(174)	.186(875)
Middle	.299(265)	.258(229)	.191(169)	.113(100)	.139(123)	.188(886)
Richer	.339(288)	.274(233)	.181(154)	.095(81)	.111(94)	.181(850)
Richest	.383(401)	.296(310)	.168(160)	.076(80)	.085(89)	.223(1048)
Residence						
Urban	.333(487)	.269(393)	.185(271)	.089(130)	.124(181)	.311(1462)
Rural	.282(912)	.248(804)	.177(572)	.121(393)	.172(558)	.689(3239)
Division						
Barisal	.299(158)	.233(123)	.172(91)	.100(53)	.195(103)	.112(528)
Chittagong	.270(266)	.240(236)	.175(172)	.114(112)	.201(198)	.209(984)
Dhaka	.285(296)	.262(272)	.192(199)	.114(118)	.148(154)	.221(1039)
Khulna	.351(223)	.296(188)	.167(106)	.102(65)	.085(54)	.135(636)
Rajshahi	.345(328)	.262(249)	.186(177)	.097(92)	.111(106)	.203(952)
Sylhet	.228(128)	.230(129)	.174(98)	.148(83)	.221(124)	.120(562)
Total	.298(1399)	.255(1197)	.179(843)	.111(523)	.157(739)	1.000(4701)

**Table 2:** Descriptive statistics of selected covariates by parity for BDHS, 2007

	1	2	3	4	>4	Overall
Family composition						
(Boy)	.514(742)					.166(742)
(Girl)	.486(702)					.157(702)
(Boy, Girl)		.527(644)				.144(644)
(Boy, Boy)		.239(292)				.065(292)
(Girl, Girl)		.235(287)				.064(287)
(>2 children)						.403(1804)
Survival status						
Died						.074(852)
Mother’s age at first birth						
Mean ± SD						18.1 ±3.2
Median						18.0
Mother’s Education						
No education	.122(137)	.187(209)	.205(230)	.177(198)	.309(346)	.251(1120)
Primary	.260(358)	.293(403)	.199(274)	.118(162)	.129(178)	.308(1375)
Secondary	.476(760)	.300(479)	.138(221)	.056(90)	.029(47)	.357(1597)
Higher	.500(188)	.348(131)	.122(46)	.019(7)	.011(4)	.084(376)
Wealth quintile						
Poorest	.192(162)	.235(198)	.204(172)	.155(131)	.214(180)	.189(843)
Poorer	.278(250)	.278(250)	.169(152)	.119(107)	.156(140)	.201(899)
Middle	.328(272)	.279(231)	.150(124)	.103(85)	.141(117)	.185(829)
Richer	.390(334)	.282(241)	.168(144)	.070(60)	.090(77)	.191(856)
Richest	.408(426)	.290(303)	.171(179)	.071(74)	.059(62)	.234(1044)
Residence						
Urban	.347(552)	.298(474)	.176(280)	.088(140)	.091(145)	.356(1591)
Rural	.310(892)	.260(749)	.170(491)	.110(317)	.150(431)	.644(2880)
Division						
Barisal	.325(187)	.276(159)	.170(98)	.108(62)	.122(70)	.129(576)
Chittagong	.326(290)	.254(226)	.165(147)	.107(95)	.147(131)	.199(889)
Dhaka	.309(299)	.289(279)	.168(162)	.119(115)	.116(112)	.216(967)
Khulna	.391(224)	.343(194)	.159(90)	.057(32)	.050(28)	.126(565)
Rajshahi	.362(278)	.295(226)	.180(138)	.077(59)	.086(66)	.172(767)
Sylhet	.239(169)	.197(139)	.192(136)	.133(94)	.239(169)	.158(707)
Total	.323(1444)	.274(1223)	.172(771)	.102(457)	.129(576)	1.000(4471)

**Table 3:** Descriptive statistics of selected covariates by parity for BDHS, 2011

	1	2	3	4	>4	Overall
Family composition						
(Boy)	.509(1228)					.179(1228)
(Girl)	.491(1185)					.172(1185)
(Boy, Girl)		.518(1064)				.155(1064)
(Boy, Boy)		.255(525)				.076(525)
(Girl, Girl)		.227(467)				.068(467)
(>2 children)						.350(2408)
Survival status						
Died						.060(961)
Mother’s age at first birth						
Mean ± SD						18.1 ±3.3
Median						18.0
Mother’s Education						
No education	.143(174)	.182(222)	.224(273)	.192(234)	.260(318)	.178(1221)
Primary	.269(553)	.296(609)	.208(428)	.124(255)	.102(210)	.299(2055)
Secondary	.449(1350)	.347(1043)	.145(435)	.039(116)	.020(60)	.437(3004)
Higher	.563(336)	.305(182)	.111(66)	.017(10)	.005(3)	.087(597)
Wealth quintile						
Poorest	.246(352)	.249(357)	.215(308)	.142(203)	.147(211)	.208(1431)
Poorer	.344(451)	.285(373)	.146(191)	.108(142)	.117(153)	.190(1310)
Middle	.364(477)	.316(414)	.166(217)	.076(100)	.079(103)	.191(1311)
Richer	.386(531)	.305(419)	.180(247)	.065(89)	.065(90)	.200(1376)
Richest	.415(602)	.340(493)	.165(239)	.056(81)	.023(34)	.211(1449)
Residence						
Urban	.400(883)	.319(704)	.157(347)	.070(155)	.054(119)	.321(2208)
Rural	.328(1530)	.290(1352)	.183(855)	.099(460)	.101(472)	.679(4669)
Division						
Barisal	.391(309)	.286(226)	.156(123)	.082(65)	.085(67)	.115(790)
Chittagong	.323(420)	.278(362)	.187(243)	.095(124)	.116(151)	.189(1300)
Dhaka	.365(427)	.292(341)	.184(215)	.085(99)	.074(87)	.170(1169)
Khulna	.423(352)	.355(295)	.143(119)	.040(33)	.040(33)	.121(832)
Rajshahi	.366(317)	.337(292)	.166(144)	.075(65)	.057(49)	.126(867)
Rangpur	.368(342)	.322(299)	.172(160)	.088(82)	.050(46)	.135(929)
Sylhet	.248(246)	.243(241)	.200(198)	.148(147)	.160(158)	.144(990)
Total	.351(2413)	.299(2056)	.175(1202)	.089(615)	.086(591)	1.000(6877)

In addition, the proportion of mothers from the poor socioeconomic class remained almost the same over the survey years (about 40 %) and the proportion of poor mothers with four or more children decreased about 10 % (from 36 to 26 %) over 2004–2011. The proportions of mothers from the urban region were 31 %, 36 %, and 32 %, respectively for the years 2004, 2007, and 2011. For the rural region, proportion of mothers with four children decreased from 17 to 10 % over the survey years. The results reveal that the percentage of mothers with more than four children decreased in all six administrative divisions over the study periods.

#### Model selection

The exploratory analysis described in the previous section provides the differentials observed in the various socioeconomic and demographic characteristics associated with the women selected in this study. To assess the effects of random frailty term in identifying important factors for correlated birth intervals of Bangladeshi women, three parametric proportional hazards models are considered: Model I (without any frailty term), Model II (mother as the only frailty term), and Model III (community or cluster as the only frailty term). The same set of covariates, namely mother’s age at first birth, parity, family composition, survival status of previous child, mother’s education, wealth index, residence, administrative division, and survey year, are considered for each of the three models. To examine the time trend of the effects of different covariates on birth interval, the interaction of survey year with mother’s age at first birth, family composition and survival status are considered for each of the three models. Moreover, both the linear and squared term of the mother’s age at first birth is considered in all three models. The Models I–III are estimated using the pooled data obtained from three BDHS surveys of the years 2004, 2007, and 2011.

Table 4 shows the estimates of the parameters and the corresponding standard errors of the three models considered. The frailty models (Models II and III) are found to fit the pooled data better than the proportional hazards model (Model I), which indicates that community and mother level unobserved random effects have a considerable impact on the distribution of birth interval. Among the frailty models, the Model II, where mother is considered as the only frailty term, is found to be the best model. One-sided likelihood ratio test (LRT) statistic is used for testing the significance of frailty parameters (mother and community).

**Table 4:** Estimates of the parameters and corresponding standard errors of the parametric PH model (Model I) and parametric frailty models (Model II–III) for birth interval of Bangladesh (BDHS 2004-2011)

Covariates	Model I		Model II		Model III	
	$\hat {\beta }$	se	$\hat {\beta }$	se	$\hat {\beta }$	se
Mother’s age at first birth						
Linear	0.055^*a*^	0.017	0.214^*a*^	0.028	0.125^*a*^	0.018
Squared	−0.002^*a*^	0.001	−0.006^*a*^	0.001	−0.004^*a*^	0.001
Parity	−0.031^*a*^	0.008	−0.156^*a*^	0.010	−0.052^*a*^	0.008
Family composition						
(Boy)	0.186^*a*^	0.025	0.157^*a*^	0.029	0.167^*a*^	0.025
(Girl)	0.246^*a*^	0.025	0.249^*a*^	0.029	0.243^*a*^	0.025
(Boy, Girl)						
(Boy, Boy)	0.001	0.031	-0.062	0.038	-0.005	0.032
(Girl, Girl)	0.149^*a*^	0.031	0.085^*b*^	0.037	0.140^*a*^	0.031
(>2 children)	0.032	0.028	0.016	0.032	0.040	0.028
Survival Status						
Dead						
Alive	−0.667^*a*^	0.021	−0.927^*a*^	0.027	−0.717^*a*^	0.022
Mother’s Education						
No education	0.282^*a*^	0.041	0.468^*a*^	0.055	0.295^*a*^	0.042
Primary	0.265^*a*^	0.040	0.367^*a*^	0.054	0.283^*a*^	0.042
Secondary	0.072^*c*^	0.040	0.081	0.052	0.091^*b*^	0.041
Higher						
Wealth quintile						
Poorest						
Poorer	−0.093^*a*^	0.018	−0.116^*a*^	0.028	−0.084^*a*^	0.019
Middle	−0.183^*a*^	0.020	−0.220^*a*^	0.029	−0.169^*a*^	0.021
Richer	−0.278^*a*^	0.021	−0.315^*a*^	0.031	−0.266^*a*^	0.023
Richest	−0.432^*a*^	0.025	−0.470^*a*^	0.036	−0.428^*a*^	0.027
Residence						
Urban						
Rural	0.071^*a*^	0.016	0.100^*a*^	0.023	0.071^*a*^	0.020
Division						
Barisal						
Chittagong	0.326^*a*^	0.023	0.396^*a*^	0.034	0.364^*a*^	0.033
Dhaka	0.029	0.024	0.046	0.035	0.062^*c*^	0.035
Khulna	−0.269^*a*^	0.028	−0.346^*a*^	0.040	−0.251^*a*^	0.039
Rajshahi	−0.205^*a*^	0.024	−0.252^*a*^	0.035	−0.173^*a*^	0.036
Sylhet	0.462^*a*^	0.024	0.634^*a*^	0.037	0.501^*a*^	0.039
Year of survey	−0.078^*a*^	0.016	−0.079^*a*^	0.022	−0.080^*a*^	0.016
Mother’s age at first birth × Year of survey	0.002^*a*^	0.001	0.002^*c*^	0.001	0.002^*a*^	0.001
Family composition × Year of survey						
(Boy) × Year	0.030^*a*^	0.008	0.041^*a*^	0.009	0.034^*a*^	0.008
(Girl) × Year	0.028^*a*^	0.008	0.047^*a*^	0.009	0.034^*a*^	0.008
(Boy, Boy) × Year	0.005	0.010	0.003	0.012	0.006	0.011
(Girl, Girl) × Year	0.012	0.010	0.029^*b*^	0.012	0.017^*c*^	0.010
(>2 children) × Year	0.001	0.007	-0.0003	0.009	0.001	0.007
Survival Status × Year of survey						
Alive × Year	−0.023^*a*^	0.007	−0.037^*a*^	0.009	−0.026^*a*^	0.008
Variance of random effects						
Community					0.055	
Mother			0.411			
Kendall’s tau, *τ*			0.170		0.027	
Baseline hazard						
Scale	0.0001		0.0001		0.0001	
Shape	2.085		2.565		2.142	
Log-likelihood	-114958		-113790		-114677	

^*a*^ *p*-Value <0.01; ^*b*^ *p*-Value <0.05; ^*c*^ *p*-Value <0.10

#### Determinants of birth intervals

The fit of Model II shows that both the linear and squared effects of the maternal age at first birth are significant, which indicate that initially the likelihood of next birth increases as the mother’s age at first birth increases and after a certain age at first birth, this likelihood starts decreasing. The estimated age at the first birth corresponding to the maximum risk of the next birth are 17.58, 17.83, and 18.08 years for 2004, 2007, and 2011, respectively. Parity is found to be significant and the likelihood of the next birth decreases as parity increases.

The birth interval tends to be significantly longer for higher educated mothers compared to mothers with primary or less education, which indicates that maternal education is an important determinant to explain the birth interval differentials. More specifically, mothers with no formal education tend to have shorter intervals between successive births (hazard ratio, HR: 1.597) than their counterparts with higher education. Moreover, mothers with primary education have 44 % more likelihood of the next birth compared to the higher educated mother. The effect of year indicates that the likelihood of successive birth is decreasing with the change of time.

Family composition is an important predictor for the distribution of the duration of birth intervals. Mothers with one child (either a boy or a girl) are more likely to have the next birth compared to the mothers with a balanced number of children (one girl and one boy) over the study period. For the three surveys, mothers with one boy (girl) have 17 % (28 %) more likelihood of the next birth compared to the mothers with one girl and one boy. There is no significant difference in the likelihood of next birth between mothers with two boys, and that of with one girl and one boy. However, mothers with two girls are more likely to have shorter intervals compared with the mothers with a boy and a girl (HR: 1.089). Moreover, a significant interaction between family composition and survey year describes the change in the birth spacing behavior of mothers having different number of children relative to the balanced number of children over time. The interaction suggests that the mothers with one child (boy or girl) and two girl children still have shorter birth interval, though the likelihood is decreasing over the years.

In all the models considered for analysing birth intervals, it has been found that the survival status of the index child has a significant effect on the likelihood of having the next child. Effect of child survival status on the timing of births is in the expected direction, and mothers with previous children alive has 60 % less likelihood of the next birth compared to the mothers who lost their previous child (Model II). A significant interaction between the survival status of the previous child and the survey year reveals that the effect of the survival of the previous child on birth spacing decreased over time.

Examining the other covariates, it is clear that mothers from the middle and rich households are less likely to have shorter birth interval compared to that of from the poor households. Moreover, the pattern of birth intervals changes according to the place of residence and administrative division. Table 4 indicates that women living in rural region have their subsequent births sooner than urban women (HR: 1.105). Women from Chittagong and Sylhet divisions have 49 % and 89 % higher likelihood of the next birth compared to Barisal division, where as women from the Khulna and Rajshahi divisions’ shows 71 % and 78 % lower likelihood of the next birth compared to the women from Barisal division. But, there is no significant difference found in the distribution of birth interval among women from Dhaka and Barisal divisions.

#### Variance of random effects

Variance of frailty can help to select an appropriate model for fitting the birth interval data. Moreover, small frailty variance indicates no significant heterogeneity among the clusters, where a large variance implies greater heterogeneity. The estimated standard errors are found to be smaller for the models without frailty (i.e. Model I) compared to that of estimated from the frailty models (Models II and III). This is expected as the frailty models account for the extra variance associated with unobserved risk factors. From our analysis, estimates of the frailty variance are 0.411 (mother level) and 0.055 (community level) indicates that the lengths of birth intervals varies largely with mother compared to the community. In addition, estimates of Kendall’s *τ* are 0.170 (mother level) and 0.027 (community level) for the frailty models.

## Discussion

Birth interval is a major determinant of fertility of a high populous country as well as an important indicator of socioeconomic development. According to BDHS (2011) policy brief, total fertility rate is declining over the time, and declination must be continued to achieve population stability in mid century with TFR 1.6 [25]. The increasing length of birth spacing will help to achieve this goal as birth interval is a determinant of fertility. From the analysis presented in previous sections, it is found that different socioeconomic and demographic factors have significant effects on the length of birth interval and some of these effects are significantly varied over time. The fits of the three models considered show that mother’s age at first birth, parity, family composition, survival status of index children, mother’s education, place of residence and division have significant influence on the length of birth interval.

Family composition is a very important determinant of birth interval because the length of birth interval depends on whether a mother has a son or a daughter. Mothers who already have a son tends to have a longer birth interval compared to that of without a son. The estimated effect of family composition on the length of birth interval is similar to the findings of Mahmood et al. [9], Maitra and Pal [14], and Setty-Venugopal and Upadhyay [15]. People often value a boy differently from a girl in social, economic and religious aspects, this could be the underlying reason of the son preference and of shorter birth spacing if a mother has no son [15, 26]. Moreover, women with the balanced composition of children have less chance of the next birth compared to others, which is comparable with the findings of Chowdhury and Bairagi [17], and Rahman and DaVanzo [18]. The interval for the next birth tends to decrease due to death of preceding child [5, 27] because couples may want to make a conscious effort to replace the lost child sooner [15], which is known as “the child replacement effect” [14].

Women’s involvement in education is one of the most beneficial measures to increase birth interval and reduce fertility [9, 19]. This protective effect of education is also in line with the findings from population report of 2002, where 38 of 51 countries showed women with no education were less likely to space births than women with education [15]. This might be due to the fact that educated women are more likely to use contraception to prolong their birth spacing [28, 29] and may have the knowledge regarding the negative effect of short birth intervals as well as benefits of small family size. Moreover, educated women are more likely to engage in occupations that may also lead to longer birth interval [15].

On the other hand, women from wealthy families have significantly long birth interval due to education and lifestyle, which is compatible with the findings of Kamal and Khalid [27]. Women from the rural areas are more likely to have shorter birth intervals compared to the women from the urban areas, which is consistent with previous studies [9, 15, 19]. According to population report 2002 [15], urban women have better access to education and employment opportunities, which may help to improve the length of birth spacing and decline the fertility.

In methodological point of view, parametric proportional hazards model without frailty underestimates the standard error of parameter estimators because the model does not consider the correlation among observations from the same cluster. In the current study, parametric frailty models are used to estimate the effects of covariates on birth interval after adjusting for the cluster variation in mother and community level. A significant variation among mothers and communities is found, which is consistent with the findings of Mahmood et al. [9]. Furthermore, R codes are developed to analyze the birth interval data obtained from three BDHS surveys using parametric frailty models because the existing R packages (e.g. parmf, survival, etc.) cannot handle such a large number of observations.

Note that the main objective of this paper is to identify potential determinants of birth intervals and examine how the impact of the important determinant changes over time after controlling the heterogeneity due to mother or community. In this paper, design weights have not been used in the analysis because objective of this paper is to estimate the effects of important determinants for birth interval, not to provide national level estimates of some indicators [30, 31].

## Conclusions

The current analysis provides evidence that socioeconomic and demographic variables do affect the distribution of birth spacing of women in Bangladesh. It could be suggested that a general social change is probably in place in Bangladesh, but we are not optimistic that this process of change has the tendency yet to induce people to opt for longer birth spacing and smaller family sizes due to smaller effect of some covariates over time. Specifically, this study emphasizes the need for gender composition and child mortality related studies in planning public health programs as per needs of the country. Further, female education also demonstrated an important protective role for increasing birth interval, so stronger strategies to ensure female education should be continued. In overall, interventions related to family planning should be stronger to keep birth interval longer and the fertility rate at the desired level. Moreover, there is a need of efficient policy for the promotion of longer birth spacing.
