# Erratum to: Trend of determinants of birth interval dynamics in Bangladesh

**DOI:** 10.1186/s12889-016-3753-y

**Published:** 2016-10-17

**Authors:** Jahidur Rahman Khan, Wasimul Bari, A. H. M. Mahbub Latif

**Affiliations:** 1 Institute of Statistical Research and Training (ISRT), University of Dhaka, Dhaka, 1000 Bangladesh; 2 Department of Statistics, Biostatistics & Informatics, University of Dhaka, Dhaka, 1000 Bangladesh; 3 Center for Clinical Epidemiology, St. Luke’s International University, 3-6-2 Tsukiji, Chuo-ku, 104-0045 Tokyo, Japan

## Erratum

During the processing of this article [1], the following typographical error occurred on page 3: , *ui* was incorrectly introduced:

1. inside the exponent in the definition of *S*_*ij*_(*t*|*X*_*ij*_, *u*_*i*_) following Eq. 3; this should read:
  $$ {S}_{ij}\left(t\left|{X}_{ij},{u}_i\right.\right)= \exp \left\{-{\mathit{\int}}_0^t{h}_{ij}\left(x\left|{X}_{ij}\right.,{u}_i\right)\kern0.1em dx\right\}= \exp \left(-{H}_{\mathrm{o}}(t){u}_i\beta \mathit{\hbox{'}}\kern0.1em {X}_{ij}\right) $$

2. inside the exponent in Eq. 4; this should read:
  $$ {L_m}_{,i}\left(\phi, \theta, \beta \right)={\displaystyle \prod_{j=1}^{n_i}{\mathit{\int}}_0^{\infty}\kern0.5em }{\left[{h}_{\mathrm{o}}\left({t}_{ij}\right){u}_i \exp \left(\beta \mathit{\hbox{'}}\kern0.1em {X}_{ij}\right)\right]}^{\delta_{ij}}\times {S}_{ij}\left({t}_{ij}\left|{X}_{ij},{u}_i\right.\right)\kern0.1em \mathit{\mathsf{g}}\kern0.1em \left({u}_i;\theta \right)d{u}_i $$

3. Twice in Eq. 5; this should read:
  $$ \begin{array}{l}{l}_m\left(\phi, \theta, \beta \right)={\displaystyle \sum_{i=1}^K\kern0.1em \left[ \log \varGamma \left\{\left(1/\theta \right)+{d}_i\right\}+{\displaystyle \sum_{j=1}^{n_i}{\delta}_{ij}}\left(\beta \mathit{\hbox{'}}\kern0.1em {X}_{ij}+ \log \left(\upgamma {t}_{ij}^{\upgamma -1}/{\alpha}^{\upgamma}\right)\right)- \log \varGamma \left(1/\theta \right)\right.}\\ {}\left.+{d}_i \log \theta -\left\{\left(1/\theta \right)+{d}_i\right\}\times \log \kern0.5em \left(1+\theta {\displaystyle \sum_{j=1}^{n_i}{\left({t}_{ij}/\alpha \right)}^{\upgamma}} \exp \left(\beta \mathit{\hbox{'}}\kern0.1em {X}_{ij}\right)\right)\right]\end{array} $$
